# Etiology of Persistent Tubo-Ovarian Abscess in Nairobi, Kenya

**DOI:** 10.1155/S1064744903000061

**Published:** 2003

**Authors:** Craig R. Cohen, Lisa Gravelle, Samwel Symekher, Peter Waiyaki, Walter E. Stamm, Julia A. Kiehlbauch

**Affiliations:** 1 Department of Obstetrics and Gynecology University of Washington Box 356460 Seattle WA 98195-6460 USA; 2 Center for Microbiology Research Kenya Medical Research Institute Kenya; 3 Department of Medical Microbiology University of Nairobi Nairobi Kenya; 4 Department of Medicine University of Washington WA USA; 5 Department of Obstetrics and Gynecology Oregon Health Sciences University Portland OR USA

## Abstract

*Objective* To study the microbial etiology of tubo-ovarian abscess (TOA).

*Methods* We recruited 11 women in Nairobi, Kenya who failed antibiotic therapy alone and required surgical drainage of a presumptive TOA. Pus from the nine abscesses and two pyosalpinges were collected and cultured for aerobic, facultative and anaerobic microorganisms.

*Results* Eleven women suspected of having a TOA were hospitalized and treated for a median of 6 days (range 3–14 days) prior to surgical drainage of the abscess. Nine (82%) specimens were culture positive. Aerobes were present in all nine specimens. Seven of the nine positive cultures (78%) were polymicrobial and five of the polymicrobial cultures contained both anaerobes and aerobes. Anaerobic Gram-negative bacilli (*Prevotella sp., Porphyromonas sp. and Bacteroides sp., Escherichia coli* ) and Streptococcus sp. (* S. viridans *and* S. agalactiae*) were the most common microorganisms isolated. *Neisseria gonorrhoeae* and *Chlamydia trachomatis* were not isolated by culture or detected by polymerase chain reaction.

*Conclusions* In Kenya, persistent TOAs are associated with endogenous flora similar to that normally found in the gastrointestinal tract.


Infect Dis Obstet Gynecol 2003;11:45–51

### Etiology of persistent tubo-ovarian abscess in Nairobi, Kenya

**Craig R. Cohen[1,2,3], Lisa Gravelle[5], Samwel Symekher[2], Peter Waiyaki[2], Walter E. Stamm[4] and Julia A. Kiehlbauch[1,2]**

[1]*Department of Obstetrics and Gynecology, University of Washington, WA*
[2]*Center for Microbiology Research, Kenya Medical Research Institute*
[3]*Department of Medical Microbiology, University of Nairobi, Nairobi, Kenya*
[4]*Department of Medicine, University of Washington, WA*
[5]*Department of Obstetrics and Gynecology, Oregon Health Sciences University, Portland, OR*

***Objective*** To study the microbial etiology of tubo-ovarian abscess (TOA).
***Methods*** We recruited 11 women in Nairobi, Kenya who failed antibiotic therapy alone and required surgical drainage of a presumptive TOA. Pus from the nine abscesses and two pyosalpinges were collected and cultured for aerobic, facultative and anaerobic microorganisms.
***Results*** Eleven women suspected of having a TOA were hospitalized and treated for a median of 6 days (range 3–14 days) prior to surgical drainage of the abscess. Nine (82%) specimens were culture positive. Aerobes were present in all nine specimens. Seven of the nine positive cultures (78%) were polymicrobial and five of the polymicrobial cultures contained both anaerobes and aerobes. Anaerobic Gram-negative bacilli (*Prevotella sp.*, *Porphyromonas sp.* and *Bacteroides sp.*, *Escherichia coli*) and *Streptococcus sp.* (*S. viridans* and *S. agalactiae*) were the most common microorganisms isolated. *Neisseria gonorrhoeae* and *Chlamydia trachomatis* were not isolated by culture or detected by polymerase chain reaction.
***Conclusions*** In Kenya, persistent TOAs are associated with endogenous flora similar to that normally found in the gastrointestinal tract.

Key words: PELVIC INFLAMMATORY DISEASE; SALPINGITIS; ANAEROBIC BACTERIA; AFRICA; ABSCESS; HIV

### INTRODUCTION

Tubo-ovarian abscess (TOA), a significant complication of pelvic inflammatory disease (PID), occurs in 3–16% of patients hospitalized with PID in the United States[1,2] and in 22% of women hospitalized with salpingitis in Nairobi, Kenya[3]. Effective management of a TOA has evolved over the past 20 years so that treatment with broad spectrum antibiotics is initiated early after initial presentation with surgical drainage reserved for patients who fail to respond[4]. Success of medical therapy alone has ranged between 67–90%; but, approximately 25% of TOAs require surgical drainage[5].

Initial studies by Altemeier found anaerobic streptococci in 92% of TOA specimens[6], and more recent investigations detected mixed aerobic and

This study was supported by the National Institutes of Health through the Sexually Transmitted Disease Clinical Trials Unit (AI75329).

Craig R. Cohen, MD, Department of Obstetrics and Gynecology, University of Washington, Box 356460, Seattle, WA 98195-6460, USA. Email: crcohen@u.washington.edu

© 2003 The Parthenon Publishing Group 

anaerobic flora in nearly all cases of TOA[4,7]. The recognition that pelvic abscesses are associated with anaerobic bacteria and are frequently polymicrobial led to improved antibiotic coverage including antibiotics like clindamycin and metronidazole that have good anaerobic activity[5]. However, patients who fail antibiotic treatment alone and require surgical drainage for a persistent TOA represent very serious and potentially life-threatening cases for medical facilities worldwide, and especially in resource-poor settings such as those found in sub-Saharan Africa. Furthermore, in sub-Saharan Africa where human immunodeficiency virus (HIV)-1 infection is common the etiology of TOA has not been established. In data from studies conducted in Africa, HIV infection has been associated with (a) a lower prevalence of gonococcal and chlamydial infection and higher odds of bacterial vaginosis (BV) in women with histologically confirmed PID[8], (b) an increased risk if a TOA among women diagnosed with acute salpingitis[3,9] and (c) an increased length of hospitalization for women diagnosed with either a TOA and/or pyosalpinx[3]. As additional understanding of the etiology of persistent TOAs could lead to more effective treatment guidelines suitable for resource-poor settings, we prospectively evaluated the microbial flora of pelvic abscesses in women hospitalized at the national referral hospital in Nairobi, Kenya who required surgical drainage after failing medical therapy alone.

### METHODS

This study protocol was approved by the Institutional Review Board for Human Subjects at the University of Washington, and by the Ethical Review Committee at Kenyatta National Hospital, Nairobi, Kenya. Procedures followed were in accordance with the ethical standards for human experimentation established by the Declaration of Helsinki of 1975, revised in 1983.

Between February and June 1999, women admitted to Kenyatta National Hospital with a presumptive diagnosis of TOA based upon pelvic examination and/or trans-abdominal ultrasound findings and who had either persistent low abdominal pain, fever and/or limited regression of a pelvic mass after receiving at least 3 days of antibiotic therapy alone were recruited. After obtaining written informed consent for collection of microbiological specimens women underwent laparotomy drainage by the hospital physician. Laparotomy, rather than a less invasive drainage procedure such as laparoscopy or ultrasound-guided drain placement was standard procedure for persistent TOA at Kenyatta National Hospital. Prior to surgery a demographic and clinical questionnaire was administered. Pus from the TOA was removed during surgery and placed in anaerobic transport media (Anaerobe Systems, San Jose, CA). Before and after surgery, patients received empiric antibiotic treatment that included intravenous (IV) penicillin 4 million IU every 6 hours and gentamicin 80 mg every 8 hours. An IV antibiotic providing activity against obligate anaerobes was not available. Oral metronidazole 400 mg every 8 hours was initiated when available in the hospital pharmacy. Patient management was directed by the physician in charge of the acute gynecology service and was not affected by participation in the study.

Study personnel were not involved in the care or diagnosis of subjects other than being present at the laparotomy procedure, and performing a short clinical/demographic questionnaire.

Following transport to the laboratory, specimens were cultured for both anaerobic and facultative organisms. Specimens were processed in an anaerobic chamber (Forma Scientific, Marietta, Ohio). All media were prepared locally from commercially available products. Each sample was inoculated onto Brucella medium enriched with vitamin K and Hemin (Oxoid, Ogdensburg, NY); Columbia agar containing colistin and nalidixic acid (CNA) (BBL, Lockeysville, MD); and Laked Kanamycin Vancomycin agar for anaerobes (Oxoid, Ogdensburg, NY); and Chocolate agar enrich with isovitalex (BBL, Lockeysville, MD); CNA agar and trypticase soy agar containing 5% sheep blood for aerobes (BBL, Lockeysville, MD). Growth of each organism type was recorded in a semi-quantitative fashion. In addition, each sample was inoculated into cooked meat medium (BBL, Cockeysville, MD). Aerobic cultures were incubated for 48 hours at 35°C in 5% $CO_2$, and anaerobic cultures incubated for 5 days at 35°C in an anaerobic chamber before they were discarded as negative. Broths were incubated anaerobically for 5 days and examined by visual inspection. Broths with visible growth were Gram-stained and subcultured to appropriate media. Each organism was identified using simple biochemical tests. Each isolate growing on anaerobic media was tested to determine if it was a facultative or obligate anaerobe. *Neisseria* (QuadFerm, Biomerieux Vitek, St. Louis, MO or Wee Tabs, Key Scientific, Round Rock, TX), *Enterobacteriaceae* (API 20E, Biomerieux Vitek, St. Louis, MO), and *Haemophilus* were identified to the species level, and staphylococci, streptococci and Gram-positive rods were identified to genus or group level as appropriate. For anaerobes, isolates of *Peptostreptococcus*, *Bacteroides*, *Fusobacterium*, *Prevotella*, *Porphytromonas* and *Bilophila/Sutterella* were identified to the species level (Wee Tab, Key Scientific, Round Rock, TX). Gram-positive rods and *Mobiluncus sp.* were identified to the genus or species level as appropriate. A polymerase chain reaction (PCR) assay (Roche Diagnostic System Inc., Somerville, NJ) was used to test for *N. gonorrhoeae* and *C. trachomatis* in a subset of specimens (Identification Nos. 106–111).

Demographic and laboratory data were collected, stored and analyzed using Excel 2000 (Microsoft, Inc., Redmond, WA).

### RESULTS

We recruited 11 subjects who had a presumptive diagnosis of a TOA and had either persistent low abdominal pain, fever and/or limited regression of a pelvic mass after receiving at least 3 days of antibiotic therapy. At surgery two women were diagnosed with a pyosalpinx and were included in the analysis. Women ranged from 18 to 39 years of age, five (45%) were married, four (36%) divorced or separated and two (18%) single, and only one currently used hormonal contraception. Gravidity ranged from 0 to 6 (median = 1), three subjects (27%) had a history of induced abortion and six subjects (55%) reported the inability to conceive for greater than 1 year, and three (27%) reported oligomenorrhea prior to development of symptoms. Ten women reported a single sexual partner during the 3 months prior to admission, while one refused to answer questions about sexual practice. History of suffering a similar disease was recalled by two (18%) participants.

All women reported acute low abdominal pain as their chief complaint and the reason for seeking hospital admission. In addition, five (45%) had a temperature ≥ 38°C, one (9%) abdominal swelling, seven (64%) abnormal vaginal discharge, six (55%) dysuria and one (9%) low back pain. Women had pain for a median of 7 days (range 1–30 days) prior to hospital admission. Most women (73%) had sought some form of outpatient medical attention before seeking hospital admission, and had been prescribed different combinations of oral antibiotics. Medical services ranged from being sold antibiotics at a local store or dispensary to a visit to a primary health care facility. Following hospitalization, intravenous antibiotics were administered for a median of 6 days (range 3–14 days) prior to surgical drainage and the collection of pus for culture (Table 1).

Overall, microorganisms were recovered from nine (82%) of 11 pelvic abscesses (seven of nine (78%) TOA and from both (100%) pyosalpinx cases). Aerobes were present in all nine (82%) and anaerobes in five (45%) culture-positive specimens. Of nine positive cultures, two (22%) were monomicrobial and seven (78%) were polymicrobial (median number of species in polymicrobial specimens = 9, range 2–21). Five of seven polymicrobial cultures contained both anaerobic and aerobic species; one TOA specimen contained two aerobes (*S. agalactiae* and *E. coli*); one pyosalpinx specimen contained *Candida sp.* other than *C. albicans* and an enteric Gram-negative rod. Table 2 contains the complete listing of microorganisms cultured from each specimen. Among anaerobes, Gram-negative bacilli (*Prevotella sp.*, *Porphyromonas sp.* and *Bacteroides sp.*) were the most common isolates. Interestingly, anaerobic species were always found together with aerobes. *E. coli* followed by the *Streptococcus sp.* (*S. viridans* and *S. agalactiae*) were the most common aerobic species detected. *N. gonorrhoeae* was not isolated by culture from any specimen, and *C. trachomatis* and *N. gonorrhoeae* were not detected by PCR in any of the six specimens tested.

**Table 1** Length of hospitalization and course of parenteral antibiotics prior to laparotomy drainage, and description of pus collection for 11 women with a persistent pelvic abscess in Nairobi, Kenya

| *Characteristic* | *Specimen identification number* | | | | | | | | | | |
|---|---|---|---|---|---|---|---|---|---|---|---|
| *Specimen* | *101* | *102* | *103* | *104* | *105* | *106* | *107* | *108* | *109* | *110* | *111* |
| Age | 26 | 33 | 25 | 35 | 30 | 38 | 39 | 32 | 18 | 23 | 28 |
| Gravity, parity | G1 P1 | G1 P1 | G3 P2 | G1 P1 | G6 P6 | G0 P0 | G5 P4 | G0 P0 | G1 P0 | G2 P1 | G2 P1 |
| Length of antibiotic course prior to surgery (days) | 10 | 3 | 4 | 6 | 6 | 5 | 6 | 14 | 12 | 4 | 7 |
| Description of abscess | POD TOA | Anterior pouch TOA without adnexal involvement | Foreign body (plastic catheter used to induce abortion); POD TOA | Bilateral pyosalpinx | Right TOA | Left pyosalpinx | POD TOA, and between bowel | Right TOA | POD TOA | Right anterior TOA | Right TOA |

POD, pouch of Douglas; TOA, tubo-ovarian abscess

### DISCUSSION

Our investigation is the first to describe in detail the microbiologic flora of pelvic abscesses in sub-Saharan Africa, where immunosuppression from HIV-1 infection is commonly associated with an increased risk of this condition in women with PID[3]. Despite prolonged IV antibiotic therapy before obtaining pus, a large assortment and number of microorganisms were recovered from persistent pelvic abscesses. The persistence of microbes in abscess contents may be due to the low redox potential in pus, poor penetration of antimicrobials, high levels of antibiotic degrading enzymes and impaired phagocytosis by neutrophils found within abscesses[10,11]. In comparison to a study of 53 women with TOA reported by investigators in San Francisco between 1970 and 1980[4], our study had a higher isolation rate (82 vs. 51%), a greater mean number of species per specimen (5.7 vs. 2.2) and a wider variety of microorganisms. Nevertheless, the spectrum of microorganisms isolated (*E. coli*, aerobic streptococci, *Bacteroides sp.* and *Peptostreptococcus sp.*, and the absence of *N. gonorrhoeae*) were remarkably similar in the two studies.

In comparison to flora cultured from women with salpingitis uncomplicated by a TOA, we noted greater numbers and variety of anaerobic and aerobic bacteria[12,13]. The flora isolated from these abscesses often included members of the *B. fragilis* group (18%) and *Enterobacteriaceae* (50%) and therefore more closely resembled gut flora. In contrast, endometrial biopsies from women with clinically suspected outpatient PID in Nairobi rarely contained *B. fragilis* group (5% of specimens) and *Enterobacteriaceae* (11% of specimens)[14].

Unfortunately, due to ethical concerns about counseling subjects regarding HIV serostatus immediately after surgery, HIV-1 testing was not included in the study protocol. However, in the earlier investigation of the effect of HIV-1 infection on acute salpingitis at the same institution, TOA were found in 33% of HIV-1 seropositive women and in 15% of HIV-1 seronegative women (odds ratio, OR 2.8, 95% confidence interval, CI 1.2–6.5), and were most common in HIV-1 seropositive women with a CD4 count < 14 (55%)[3]. It is possible that impaired immune defenses in the genital tract and the increased prevalence of BV[8]

**Table 2** Prevalence of facultative and anaerobic bacteria found in pelvic abscesses (tubo-ovarian abscess (TOA) and psyosalpinx (Pyo)) from women undergoing laparotomy drainage in Nairobi, Kenya

| | *Specimen identification number* | | | | | | | | | | | | |
|---|---|---|---|---|---|---|---|---|---|---|---|---|---|
| | *101* | *102* | *103* | *104* | *105* | *106* | *107* | *108* | *109* | *110* | *111* | | |
| *Characteristic* | *TOA* | *TOA* | *TOA* | *Pyo* | *TOA* | *Pyo* | *TOA* | *TOA* | *TOA* | *TOA* | *TOA* | *Total number* | *% of specimens* |
| **Anaerobes** | 5 | 0 | 2 | 0 | 0 | 0 | 12 | 0 | 7 | 0 | 9 | **5** | **45** |
| Gram-positive cocci | 0 | 0 | 1 | 0 | 0 | 0 | 4 | 0 | 3 | 0 | 0 | **3** | **27** |
| *Peptostreptococcus sp.* | 0 | 0 | 0 | 0 | 0 | 0 | 4 | 0 | 3 | 0 | 0 | **2** | **18** |
| *Peptostrep. anaerobius* | | | | | | | + | | + | | | **2** | **18** |
| *Peptostrep. magnus* | | | | | | | + | | | | | **1** | **9** |
| *Peptostrep. prevotti* | | | | | | | + | | + | | | **2** | **18** |
| *Peptostrep. sp.* | | | | | | | + | | | | | **1** | **9** |
| *Peptostrep. tetradius* | | | | | | | | | + | | | **1** | **9** |
| *Staphylococcus saccharolyticus* | | | + | | | | | | | | | **1** | **9** |
| Gram-positive bacilli | 1 | 0 | 0 | 0 | 0 | 0 | 0 | 0 | 0 | 0 | 3 | **2** | **18** |
| *Actinomyces meyeri* | | | | | | | | | | | + | **1** | **9** |
| *Eubacterium lentum* | + | | | | | | | | | | + | **2** | **18** |
| *Lactobacillus acidophilus/casei* | | | | | | | | | | | + | **1** | **9** |
| *Veillonella sp.* | | | | | | | + | | | | | **1** | **9** |
| Gram-negative bacilli | 4 | 0 | 1 | 0 | 0 | 0 | 7 | 0 | 4 | 0 | 6 | **5** | **45** |
| *Bacteroides sp.* | 0 | 0 | 0 | 0 | 0 | 0 | 2 | 0 | 0 | 0 | 5 | **2** | **18** |
| *B. fragilis* group | 0 | 0 | 0 | 0 | 0 | 0 | 1 | 0 | 0 | 0 | 5 | **2** | **18** |
| *B. cacae* | | | | | | | + | | | | + | **2** | **18** |
| *B. distasonis* | | | | | | | | | | | + | **1** | **9** |
| *B. fragilis* | | | | | | | | | | | + | **1** | **9** |
| *B. merdae* | | | | | | | | | | | + | **1** | **9** |
| *B. vulgatus* | | | | | | | | | | | + | **1** | **9** |
| Non-*B. fragilis* group | | | | | | | 1 | | | | | **1** | **9** |
| *B. ureolyticus* | | | | | | | + | | | | | **1** | **9** |
| *Bilophila wadsworthia* | | | | | | | | | | | + | **1** | **9** |
| *Porphyromonas sp.* | 2 | 0 | 0 | 0 | 0 | 0 | 2 | 0 | 2 | 0 | 0 | **3** | **27** |
| *Porphyromonas endodontalis* | + | | | | | | + | | + | | | **3** | **27** |
| *Porphyromonas sp.* | + | | | | | | + | | + | | | **3** | **27** |
| *Prevotella sp.* | 2 | 0 | 1 | 0 | 0 | 0 | 3 | 0 | 2 | 0 | 0 | **4** | **36** |
| Non-pigmented prevotella sp. | 0 | 0 | 1 | 0 | 0 | 0 | 1 | 0 | 1 | 0 | 0 | **3** | **27** |
| *Prevotella bivia* | | | + | | | | | | | | | **1** | **9** |
| *Prevotella buccae* | | | | | | | + | | | | | **1** | **9** |
| *Prevotella buccalis/veroralis* | | | | | | | | | + | | | **1** | **9** |
| Pigmented Prevotella sp. | 2 | 0 | 0 | 0 | 0 | 0 | 2 | 0 | 1 | 0 | 0 | **3** | **27** |
| *Prevotella corporis* | + | | | | | | | | | | | **1** | **9** |
| *Prevotella denticola* | | | | | | | + | | + | | | **2** | **18** |
| *Prevotella loescheii* | + | | | | | | | | | | | **1** | **9** |
| *Prevotella malaninogenica* | | | | | | | + | | | | | **1** | **9** |

*Continued*

in immunosuppressed HIV-1 seropositive women may increase the risk for higher concentrations of microorganisms to pass through the lower genital tract into the endometrium, Fallopian tubes and peritoneal cavity to generate a complicated microbial flora prone to abscess formation. Although we are uncertain as to how well the organisms isolated after prolonged antimicrobial therapy represent the bacteria present during the formation of the abscess, the microorganisms

**Table 2** *continued*

| | *Specimen identification number* | | | | | | | | | | | | |
|---|---|---|---|---|---|---|---|---|---|---|---|---|---|
| | *101* | *102* | *103* | *104* | *105* | *106* | *107* | *108* | *109* | *110* | *111* | *Total* | *% of* |
| *Characteristic* | *TOA* | *TOA* | *TOA* | *Pyo* | *TOA* | *Pyo* | *TOA* | *TOA* | *TOA* | *TOA* | *TOA* | *number* | *specimens* |
| **All aerobes** | 4 | 0 | 2 | 2 | 2 | 1 | 2 | 0 | 4 | 1 | 12 | **9** | **82** |
| Candida sp. other than *C. albicans* | | | | + | | | | | | | + | **2** | **18** |
| *Neisseria gonorrhoeae** | | | | | | | | | | | | **0** | **0** |
| *Chlamydia trachomatis*† | * | * | * | * | * | | | | | | | **0** | **0** |
| Aerobic bacteria | 4 | 0 | 2 | 1 | 2 | 1 | 2 | 0 | 4 | 1 | 11 | **9** | **82** |
| Gram-positive cocci | 3 | 0 | 1 | 0 | 1 | 0 | 0 | 0 | 2 | 1 | 4 | **6** | **55** |
| *Staphylococcus sp.* | 1 | 0 | 0 | 0 | 0 | 0 | 0 | 0 | 0 | 1 | 2 | **3** | **27** |
| *Staph. aureus* | | | | | | | | | | | + | **1** | **9** |
| *Staph.* coagulase negative | + | | | | | | | | | + | + | **3** | **27** |
| *Streptococcus sp.* | 2 | 0 | 1 | 0 | 1 | 0 | 0 | 0 | 2 | 0 | 2 | **5** | **45** |
| Strep. not A, B or D (β heme) | | | | | | | | | + | | | **1** | **9** |
| *Strep.* Group B | + | | | | + | | | | | | | **2** | **18** |
| *S. viridans* | + | | + | | | | | | + | | + | **4** | **36** |
| *Enterococcus sp.* | | | | | | | | | | | + | **1** | **9** |
| Gram-positive bacilli | 0 | 0 | 1 | 0 | 0 | 0 | 2 | 0 | 1 | 0 | 2 | **4** | **36** |
| *Actinomyces sp.* | | | | | | | + | | | | + | **2** | **18** |
| *Bacillus sp.* | | | + | | | | | | | | | **1** | **9** |
| *Corynebacterium sp.* | | | | | | | | | + | | | **1** | **9** |
| *Lactobacillus sp.* | | | | | | | + | | | | + | **2** | **18** |
| Gram-negative bacilli | 1 | 0 | 0 | 1 | 1 | 1 | 0 | 0 | 1 | 0 | 5 | **6** | **55** |
| *Enterobacteriaceae* | 1 | 0 | 0 | 1 | 1 | 1 | 0 | 0 | 1 | 0 | 4 | **6** | **55** |
| *Escherichia coli* | | | | + | + | + | | | + | | + | **5** | **45** |
| *Klebsiella ozaenae* | | | | | | | | | | | + | **1** | **9** |
| *Klebsiella pneumoniae* | | | | | | | | | | | + | **1** | **9** |
| *Proteus mirabilis* | | | | | | | | | | | + | **1** | **9** |
| *Salmonella sp.* | + | | | | | | | | | | | **1** | **9** |
| *Pseudomonas sp.* | | | | | | | | | | | + | **1** | **9** |
| **Bacteria per specimen** (n) | **9** | **0** | **4** | **1** | **2** | **1** | **14** | **0** | **11** | **1** | **20** | **9** | **82** |

*N. gonorrhoeae culture was performed on all specimens, and PCR was done on specimens 106–111; †C. trachomatis PCR was done on specimens 106–111

isolated implicate endogenous gastrointestinal flora rather than either normal vaginal or BV-associated flora. If future studies show that endogenous gastrointestinal flora is responsible for a large percentage of PID and TOA in HIV-1-infected immunosuppressed women, we may need to alter our approach for prevention of these diseases in HIV-1-endemic populations. In addition, our findings clearly indicate the importance of early initiation of antibiotics with excellent coverage against obligate anaerobes for suspected cases of TOA and pyosalpinx in this setting.

### REFERENCES

1. Nebel WA, Lucas WE. Management of tubo-ovarian abscess. *Obstet Gynecol* 1968;32:382–6
2. Clark JFJ, Moore-Hines S. A study of tubo-ovarian abscess at Howard University Hospital (1965 through 1975). *J Nat Med Assoc* 1979;71:1109–11
3. Cohen CR, Sinei S, Reilly M, *et al.* HIV and acute pelvic inflammatory disease: a laparoscopic study. *J Infect Dis* 1998;178:1352–8
4. Landers DV, Sweet RL. Tubo-ovarian abscess: contemporary approach and management. *Rev Infect Dis* 1983;5:876–84
5. Wiesenfeld HC, Sweet RL. Progress in the management of tubo-ovarian abscesses. *Am J Obstet Gynecol* 1993;36:433–44
6. Altemeir WA. The anaerobic streptococci in tubo-ovarian abscess. *Am J Obstet Gynecol* 1940; 39:1038–42
7. Sweet RL. Anaerobic infections in the female genital tract. *Am J Obstet Gynecol* 1975;122: 891–901
8. Bukusi E, Cohen CR, Stevens C, *et al.* Effect of HIV-1 infection on microbial etiology and efficacy of ambulatory oral therapy of pelvic inflammatory disease. *Am J Obstet Gynecol* 1999;181:1374–81
9. Kamenga MC, De Cock KM, St. Louis ME, *et al.* The impact of human immunodeficiency virus infection on pelvic inflammatory disease: a case–control study in Abidjan, Ivory Coast. *Am J Obstet Gynecol* 1995;172:919–25
10. Galandiuk S, Appel S, Polk HC. A biological basis for altered host defences in surgically infected abscesses. *Ann Surg* 1993;217:624–32
11. Ellis M, Gupta S, Galant S, *et al.* Impaired neutrophil function in patients with AIDS or AIDS-related complex: a comprehensive evaluation. *J Infect Dis* 1988;158:1268–76
12. Hillier SL, Kiviat NB, Hawes SE, *et al.* Role of bacterial vaginosis-associated microorganisms in endometritis. *Am J Obstet Gynecol* 1996;175: 435–41
13. Arredondo JL, Diaz V, Gaitan E, *et al.* Oral clindamycin and ciprofloxacin versus intramuscular ceftriazone and oral doxycycline in the treatment of mild-to-moderate pelvic inflammatory disease in outpatients. *Clin Infect Dis* 1997; 24:170–8
14. Cohen CR, Kiehlbauch JA, Meier A, *et al.* Impact of HIV-1 infection on microbiologic etiology of symptomatic plasma cell endometritis in Nairobi, Kenya: evidence for an important role for anaerobes. 13th World AIDS Conference, Durban, South Africa, July 2000 (Abstr. Mo3266)

RECEIVED 06/14/02; ACCEPTED 12/05/02
